# Pd/C-catalyzed, copper-free Sonogashira coupling: one-pot synthesis of 1-aryl-4-(2-phenylethynyl)[1,2,4]triazolo[4,3-*a*]quinoxalines in water

**DOI:** 10.1007/s00706-012-0904-4

**Published:** 2013-02-14

**Authors:** Mohammad Bakherad, Saeideh Jajarmi

**Affiliations:** School of Chemistry, Shahrood University of Technology, Shahrood, Iran

**Keywords:** Palladium catalyst, Triazoloquinoxaline, Sonogashira reaction, Phenylacetylene

## Abstract

**Abstract:**

Copper-free, Pd/C-catalyzed, one-pot reaction of 2,3-dichloroquinoxaline with hydrazine hydrate, bromine, phenylacetylene, and a variety of aldehydes provides an efficient and direct method for the preparation of 1-aryl-4-(2-phenylethynyl)[1,2,4]triazolo[4,3-*a*]quinoxalines in water at 70 °C. This methodology involves the use of inexpensive reagents or catalysts, and thus permits a new and practical access to triazolo[4,3-*a*]quinoxalines.

**Graphical abstract:**

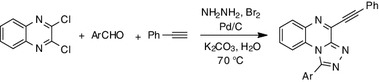

## Introduction

Quinoxaline and its derivatives show a broad spectrum of biological activities including anti-tumor [1], anti-viral [2], anti-tuberculosis [3], and anti-inflammatory [4] activities, and, hence, are an important class of nitrogen-containing heterocycles, and useful intermediates in organic synthesis [5]. Furthermore, triazolo[4,3-*a*]quinoxalines have been shown to be potent and selective adenosine receptor ligands [6].

The Sonogashira reaction catalyzed by palladium and copper is a powerful and straightforward method for the construction of arylated internal alkyne compounds [7–9]. These are important intermediates in organic synthesis and for the preparation of natural products [10], biologically active molecules [11], molecular electronics [12], and polymers [13].

Typically, these coupling-cyclization reactions are carried out using a palladium catalyst [e.g., Pd(PPh_3_)_4_, (PPh_3_)_2_PdCl_2_, etc.] and a copper salt as the co-catalyst in the presence of an amine base. The use of palladium on charcoal as a catalytic system for the efficient Sonogashira coupling of aryl halides with terminal alkynes has also been reported [14–17]. Compared to the frequently used expensive palladium catalysts [e.g., Pd(PPh_3_)_4_, (PPh_3_)_2_PdCl_2_, etc.], Pd/C-based methods have an economic advantage, and, hence, remain attractive for large or industrial scale preparations.

In recent years, a variety of modifications have been reported for this reaction, and significant progress has been made [18–22]. The most important modification is the elimination of the copper salt [23–27] since it can induce Glaser-type homocoupling of terminal alkynes in the presence of oxidants or air [28–30]. One of the powerful tools used to combine economic aspects with the environmental concerns is performing organic reactions in water; this strategy consists of two or more synthetic steps, which are carried out in water as a cheap, nontoxic, and environmentally friendly solvent, in a one-step reaction, without isolation of any intermediate, thus reducing time, and saving money, energy, and raw materials [31–33]. Several examples of Pd-catalyzed Sonogashira reactions in aqueous media have been reported [34–37].

Recently, our group has prepared pyrrolo[2,3-*b*]quinoxaline derivatives under mild conditions in one-pot reactions catalyzed by Pd–Cu [38–40]. We attempted to make this overall approach more attractive synthetically by examining the preparation of triazolo[4,3-*a*]quinoxalines using a one-pot reaction of 2,3-dichloroquinoxaline, hydrazine, aldehydes, bromine, and phenylacetylene catalyzed by Pd/C without the use of copper as a co-catalyst in water.

## Results and discussion

We reported the synthesis of new derivatives of triazolo[4,3-*a*]quinoxalines via 10 % palladium/charcoal activated (Merck catalyst) catalyzed one-pot reaction of 2,3-dichloroquinoxaline, hydrazine, a suitable aldehyde, bromine, and phenylacetylene at 70 °C by employing water as the reaction medium. The use of water as a reaction medium represents remarkable benefits since this green solvent is highly polar and, therefore, immiscible with most organic compounds; moreover, the water-soluble by-products reside, and separation of the organic materials is thus easy.

Retrosynthetic analysis indicated that the desired 1-aryl-4-(2-phenylethynyl)[1,2,4]triazolo[4,3-*a*]quinoxalines can also be synthesized from 2,3-dichloroquinoxaline, hydrazine, a suitable aldehyde, and phenylacetylene as the starting materials (Scheme 1).

**Figure:**
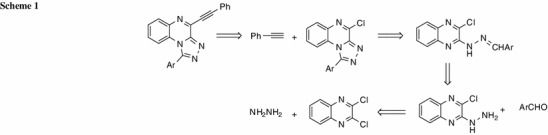
Scheme 1

For optimization of the reaction conditions, we studied the effect of various reaction parameters including the base, palladium catalyst, and temperature on the outcome of the copper-free reaction of 2,3-dichloroquinoxaline (1 equiv.) with hydrazine hydrate (2 equiv.), benzaldehyde (1 equiv.), bromine (1 equiv.), and phenylacetylene (1 equiv.) in the presence of 10 % Pd/C (5 mol %) and K_2_CO_3_ (2 equiv.) in water at 70 °C (Table 1).

**Table 1:** Effect of base and catalyst on the heterocyclization during the reaction of 2,3-dichloroquinoxaline (**1**) with hydrazine hydrate, bromine, benzaldehyde, and phenylacetylene^a^

Entry	Catalyst/mol%	Base	Yield/%^b^
1	10 % Pd/C (5)	Cs_2_CO_3_	75
2	10 % Pd/C (5)	K_2_CO_3_	82
3	10 % Pd/C (5)	KOH	80
4	10 % Pd/C (5)	Na_2_CO_3_	78
5	10 % Pd/C (5)	Et_3_N	58
6	10 % Pd/C (5)	DIEA^c^	42
7	10 % Pd/C (5)	Pyridine	54
8	10 % Pd/C (5)	Piperidine	60
9	PdCl_2_ (5)/PPh_3_ (10)	K_2_CO_3_	50
10	PdCl_2_(PPh_3_)_2_ (5)	K_2_CO_3_	70
11	Pd(dba)_2_ (5)	K_2_CO_3_	74
12	PdCl_2_ (5)	K_2_CO_3_	45
13	10 % Pd/C (3)	K_2_CO_3_	55
14	10 % Pd/C (10)	K_2_CO_3_	82
15^d^	10 % Pd/C (5)	K_2_CO_3_	35

^a^Reaction conditions: **1** (1.0 mmol), hydrazine hydrate (2.0 mmol), **2a** (1.0 mmol), bromine (1.0 mmol), phenylacetylene (1.0 mmol), K_2_CO_3_ (2.0 mmol), 70 °C, 12 h

^b^Isolated yields

^c^Diisopropylethylamine

^d^Reaction at 25 °C

The reaction was influenced significantly by the base employed. It worked very well when inorganic bases such as K_2_CO_3_ and KOH were used (entries 2 and 3), with the best result obtained in the case of potassium carbonate. Two of the organic bases used, triethylamine and piperidine, also gave good yields (entries 5 and 8). It was found that while PdCl_2_(PPh_3_)_2_, PdCl_2_/PPh_3_, PdCl_2_, and Pd_2_(dba)_3_·CHCl_3_ all catalyzed the reaction without the aid of any surfactant (entries 9–12), 10 % Pd/C turned out to be the best catalyst in terms of yields (entry 2). When the catalyst loading was decreased to 3 mol %, the yield of **4a** was 55 % (entry 13). Increasing the amount of palladium catalyst did not increase the yield (entry 14). Decreasing the temperature to 25 °C caused the yield to decrease to 35 % (entry 15).

In order to examine the scope of this reaction, compound **1** was reacted with hydrazine, various aldehydes **2**, bromine, and phenylacetylene in water at 70 °C to triazolo[4,3-*a*]quinoxalines in moderate to good yields (Table 2). When the less reactive acetylene, 1-hexyne, 1-octyne, and propargyl alcohol were used, Sonogashira coupling could not be achieved. The reactions were carried out under an argon atmosphere, and water was degassed prior to use.

**Table 2:** Synthesis of triazolo[4,3-*a*]quinoxalines **4a**–**4k** ^a^

Entry	Ar	Product	Yield/%^b^
1	C_6_H_5_	**4a**	82
2	2-NO_2_–C_6_H_4_	**4b**	94
3	3-NO_2_–C_6_H_4_	**4c**	74
4	4-NO_2_–C_6_H_4_	**4d**	94
5	2-F–C_6_H_4_	**4e**	76
6	4-F–C_6_H_4_	**4f**	85
7	2-Cl–C_6_H_4_	**4g**	83
8	4-Cl–C_6_H_4_	**4h**	86
9	2,6-Cl_2_–C_6_H_3_	**4i**	75
10	4-CH_3_–C_6_H_4_	**4j**	80
11	4-CH_3_O–C_6_H_4_	**4k**	81

^a^Reaction conditions: **1** (1.0 mmol), hydrazine hydrate (2.0 mmol), **2a**–**2k** (1.0 mmol), phenylacetylene (1.0 mmol), bromine (1.0 mmol), K_2_CO_3_ (2.0 mmol), 10 % Pd/C (5 mol %), 5 cm^3^ degassed water, 70 °C, 10 h

^b^Isolated yields

Mechanistically, the formation of triazolo[4,3-*a*]quinoxalines **4** involves the following steps (Scheme 2): (i) formation of 1-aryl-4-chloro[1,2,4]triazolo[4,3-*a*]quinoxalines from the cyclization of intermediate **A** catalyzed by bromine [41]; and (ii) Sonogashira coupling reaction of **3** with phenyl acetylene.

**Figure:**
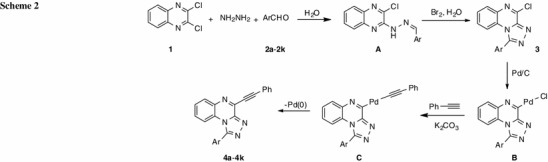
Scheme 2

## Conclusion

In conclusion, the chemistry outlined here provides a simple, efficient, clean, and straightforward method for the synthesis of triazolo[4,3-*a*]quinoxaline derivatives via the copper-free, Pd/C-catalyzed reaction of readily available dichloroquinoxaline with hydrazine, bromine, phenylacetylene, and a variety of aldehydes in water. This methodology does not involve the use of expensive reagents or catalysts, and thus permits a new and practical access to triazolo[4,3-*a*]quinoxalines.

## Experimental

All the compounds (phenylacetylene, aldehydes, Pd/C) were obtained from Merck. The reagents used were purchased from commercial suppliers, and used without further purification. Merck silica gel 60 was used for chromatography (230–400 mesh). The IR spectra were obtained as KBr pellets in the range of 400–4,000 cm^−1^ on a Shimadzu Model 460 spectrophotometer. ^1^H NMR spectra were recorded at 400 MHz, and ^13^C NMR spectra were recorded at 100 MHz in DMSO-*d*_*6*_. The chemical shifts are reported in ppm (*δ*), and referenced to the residual proton signal for DMSO-*d*_*6*_ (2.49 ppm). The coupling constants (*J*) are reported in Hz. Mass spectrometric data (MS) were obtained by electron ionization (EI, 70 eV) or electrospray ionization (ESI).

### *Typical procedure for the synthesis of 1*-*aryl*-*4*-*(2*-*phenylethynyl)[1,2,4]triazolo[4,3*-*a]quinoxalines****4a***–***4k***

A mixture of 199 mg 2,3-dichloroquinoxaline (1.0 mmol), 0.1 cm^3^ hydrazine hydrate (99 %, 2.0 mmol), a suitable aldehyde (1.0 mmol), and 0.05 cm^3^ bromine (1.0 mmol) were stirred in 5 cm^3^ H_2_O at room temperature under an argon atmosphere for 30 min. Phenylacetylene (0.11 cm^3^, 1 mmol), 53 mg Pd/C (10 %, 0.05 mmol), and 276 mg K_2_CO_3_ (2.0 mmol) were added, and the resulting solution was heated at 70 °C for 12 h, and then cooled to room temperature. The reaction mixture was filtered, and the filtrate was concentrated in vacuo. The residue was purified by chromatography, eluting with CHCl_3_/CH_3_OH (98:2) to afford the pure product (Table 2).

#### *1*-*Phenyl*-*4*-*(2*-*phenylethynyl)[1,2,4]triazolo[4,3*-*a]quinoxaline* (**4a**, C_23_H_14_N_4_)

White solid; yield 283 mg (82 %); m.p.: 280–282 °C; *R*_*f*_ = 0.53; IR (KBr): $\overline{\nu }$ = 2,200, 1,650 cm^−1^; ^1^H NMR (400 MHz, DMSO-*d*_6_): *δ* = 7.32–7.42 (m, 4H), 7.45–7.55 (m, 4H), 7.67 (d, *J* = 4.8 Hz, 1H), 7.81 (d, *J* = 4.1 Hz, 1H), 7.86–7.92 (m, 3H), 8.05–8.08 (m, 1H) ppm; ^13^C NMR (100 MHz, DMSO-*d*_6_): *δ* = 85.26, 96.37, 123.80, 127.55, 127.85, 128.11, 128.21, 128.70, 128.95, 129.22, 129.55, 129.76, 130.45, 132.10, 132.45, 138.92, 142.17, 145.11, 149.12 ppm; MS (EI, 70 eV): *m/z* (%) = 346 ([M^+^], 100), 269 (52), 245 (22), 167 (16); HRMS (ESI): *m/z* = 346.1201.

#### *1*-*(2*-*Nitrophenyl)*-*4*-*(2*-*phenylethynyl)[1,2,4]triazolo[4,3*-*a]quinoxaline* (**4b**, C_23_H_13_N_5_O_2_)

White solid; yield 367 mg (94 %); m.p.: 284–286 °C; *R*_*f*_ = 0.55; IR (KBr): $\overline{\nu }$ = 2,200, 1,600, 1,540, 1,340 cm^−1^; ^1^H NMR (400 MHz, DMSO-*d*_6_): *δ* = 7.56–7.63 (m, 3H), 7.73–7.75 (m, 1H), 7.80–7.87 (m, 3H), 7.95 (dd, *J* = 6.4, 2.0 Hz, 2H), 8.05–8.10 (m, 2H), 8.16–8.19 (m, 2H) ppm; ^13^C NMR (100 MHz, DMSO-*d*_6_): *δ* = 85.39, 96.45, 121.36, 122.72, 127.45, 128.25, 128.35, 128.47, 128.66, 129.42, 129.58, 129.68, 131.36, 132.45, 132.60, 135.54, 142.36, 145.78, 149.25 ppm; MS (EI, 70 eV): *m/z* (%) = 391 ([M^+^], 100), 345 (32), 314 (51), 290 (21), 169 (24); HRMS (ESI): *m/z* = 391.1037.

#### *1*-*(3*-*Nitrophenyl)*-*4*-*(2*-*phenylethynyl)[1,2,4]triazolo[4,3*-*a]quinoxaline* (**4c**, C_23_H_13_N_5_O_2_)

White solid; yield 289 mg (74 %); m.p.: 274–276 °C; *R*_*f*_ = 0.52; IR (KBr): $\overline{\nu }$ = 2,230, 1,610, 1,530, 1,340 cm^−1^; ^1^H NMR (400 MHz, DMSO-*d*_6_): *δ* = 7.28 (s, 1H), 7.35–7.40 (m, 2H), 7.46–7.52 (m, 3H), 7.54–7.60 (m, 3H), 7.64–7.71 (m, 3H), 8.03–8.06 (m, 1H) ppm; ^13^C NMR (100 MHz, DMSO-*d*_6_): *δ* = 85.26, 96.35, 121.10, 122.45, 127.52, 128.45, 129.26, 129.45, 129.64, 129.88, 130.20, 131.36, 132.25, 132.45, 142.36, 145.56, 148.92, 149.55 ppm; MS (EI, 70 eV): *m/z* (%) = 391 ([M^+^], 100), 345 (35), 314 (43), 290 (26), 167 (21); HRMS (ESI): *m/z* = 391.1042.

#### *1*-*(4*-*Nitrophenyl)*-*4*-*(2*-*phenylethynyl)[1,2,4]triazolo[4,3*-*a]quinoxaline* (**4d**, C_23_H_13_N_5_O_2_)

White solid; yield 367 mg (94 %); m.p.: 293–295 °C; *R*_*f*_ = 0.54; IR (KBr): $\overline{\nu }$ = 2,210, 1,620, 1,540, 1,320 cm^−1^; ^1^H NMR (400 MHz, DMSO-*d*_6_): *δ* = 7.40 (d, *J* = 8.4 Hz, 1H), 7.55–7.61 (m, 4H), 7.68–7.71 (m, 1H), 7.80–7.86 (m, 2H), 8.11–8.18 (m, 3H), 8.55 (d, *J* = 8.2 Hz, 2H) ppm; ^13^C NMR (100 MHz, DMSO-*d*_6_): *δ* = 84.95, 96.35, 116.61, 120.60, 124.75, 126.02, 128.50, 129.68, 130.72, 131.29, 132.14, 132.85, 134.87, 135.97, 136.09, 145.33, 148.53, 149.46 ppm; MS (EI, 70 eV): *m/z* (%) = 391 ([M^+^], 100), 345 (40), 314 (47), 290 (18), 168 (29); HRMS (ESI): *m/z* = 391.1047.

#### *1*-*(2*-*Fluorophenyl)*-*4*-*(2*-*phenylethynyl)[1,2,4]triazolo[4,3*-*a]quinoxaline* (**4e**, C_23_H_13_FN_4_)

White solid; yield 276 mg (76 %); m.p.: 305–307 °C; *R*_*f*_ = 0.50; IR (KBr): $\overline{\nu }$ = 2,250, 1,570 cm^−1^; ^1^H NMR (400 MHz, DMSO-*d*_6_): *δ* = 7.20 (s, 1H), 7.35–7.43 (m, 4H), 7.57–7.64 (m, 4H), 7.82–7.86 (m, 2H), 8.21 (d, *J* = 8.0 Hz, 2H) ppm; ^13^C NMR (100 MHz, DMSO-*d*_6_): *δ* = 85.80, 96.42, 116.62, 122.26, 122.92, 124.85, 127.70, 128.37, 128.81, 129.21, 130.35, 130.56, 133.51, 133.65, 141.52, 146.23, 146.56, 149.55, 160.42 ppm; MS (EI, 70 eV): *m/z* (%) = 364 ([M^+^], 100), 345 (27), 287 (62), 263 (42), 167 (25); HRMS (ESI): *m/z* = 364.1152.

#### *1*-*(4*-*Fluorophenyl)*-*4*-*(2*-*phenylethynyl)[1,2,4]triazolo[4,3*-*a]quinoxaline* (**4f**, C_23_H_13_FN_4_)

White solid; yield 309 mg (85 %); m.p.: 313–315 °C; *R*_*f*_ = 0.51; IR (KBr): $\overline{\nu }$ = 2,230, 1,590 cm^−1^; ^1^H NMR (400 MHz, DMSO-*d*_6_): *δ* = 7.20 (s, 1H), 7.35–7.41 (m, 2H), 7.55–7.62 (m, 2H), 7.78–7.82 (m, 2H), 7.90–7.98 (m, 4H), 8.15–8.19 (m, 2H) ppm; ^13^C NMR (100 MHz, DMSO-*d*_6_): *δ* = 85.82, 96.44, 116.36, 116.45, 122.32, 126.22, 127.32, 128.24, 128.55, 128.74, 128.92, 129.20, 130.35, 130.47, 132.10, 132.21, 142.30, 145.25, 149.25, 162.92 ppm; MS (EI, 70 eV): *m/z* (%) = 364 ([M^+^], 100), 345 (32), 287 (67), 263 (35), 167 (29); HRMS (ESI): *m/z* = 364.1144.

#### *1*-*(2*-*Chlorophenyl)*-*4*-*(2*-*phenylethynyl)[1,2,4]triazolo[4,3*-*a]quinoxaline* (**4g**, C_23_H_13_ClN_4_)

White solid; yield 315 mg (83 %); m.p.: 276–278 °C; *R*_*f*_ = 0.56; IR (KBr): $\overline{\nu }$ = 2,250, 1,600 cm^−1^; ^1^H NMR (400 MHz, DMSO-*d*_6_): *δ* = 7.35 (s, 1H), 7.43–7.52 (m, 4H), 7.60–7.70 (m, 4H), 8.06–8.12 (m, 2H), 8.40 (d, *J* = 8.0 Hz, 2H) ppm; ^13^C NMR (100 MHz, DMSO-*d*_6_): *δ* = 85.65, 96.53, 123.42, 127.53, 127.65, 128.21, 128.32, 128.42, 128.65, 129.25, 129.64, 130.36, 130.54, 132.25, 132.36, 132.46, 138.65, 142.35, 145.65, 149.58 ppm; MS (EI, 70 eV): *m/z* (%) = 382 ([M^+^][^37^Cl], 31), 380 ([M^+^][^35^Cl], 100), 345 (35), 303 (57), 279 (21), 167 (25); HRMS (ESI): *m/z* = 380.0850.

#### *1*-*(4*-*Chlorophenyl)*-*4*-*(2*-*phenylethynyl)[1,2,4]triazolo[4,3*-*a]quinoxaline* (**4h**, C_23_H_13_ClN_4_)

White solid; yield 326 mg (86 %); m.p.: 289–291 °C; *R*_*f*_ = 0.55; IR (KBr): $\overline{\nu }$ = 2,240, 1,600 cm^−1^; ^1^H NMR (400 MHz, DMSO-*d*_6_): *δ* = 7.41–7.45 (m, 1H), 7.55–7.63 (m, 4H), 7.65–7.69 (m, 1H), 7.71–7.77 (m, 3H), 7.85 (d, *J* = 8.0 Hz, 2H), 8.11–8.18 (m, 2H) ppm; ^13^C NMR (100 MHz, DMSO-*d*_6_): *δ* = 85.01, 96.20, 116.25, 120.63, 126.18, 127.49, 128.34, 129.68, 129.85, 130.60, 131.27, 132.45, 132.83, 136.03, 136.46, 136.80, 145.11, 149.14 ppm; MS (EI, 70 eV): *m/z* (%) = 382 ([M^+^][^37^Cl], 32), 380 ([M^+^][^35^Cl], 100), 345 (30), 303 (62), 279 (26), 168 (20); HRMS (ESI): *m/z* = 380.0850.

#### *1*-*(2,6*-*Dichlorophenyl)*-*4*-*(2*-*phenylethynyl)[1,2,4]triazolo[4,3*-*a]quinoxaline* (**4i**, C_23_H_12_Cl_2_N_4_)

White solid; yield 310 mg (75 %); m.p.: 267–269 °C; *R*_*f*_ = 0.57; IR (KBr): $\overline{\nu }$ = 2,210, 1,620 cm^−1^; ^1^H NMR (400 MHz, DMSO-*d*_6_): *δ* = 7.20 (s, 1H), 7.40–7.48 (m, 4H), 7.55–7.62 (m, 3H), 7.75–7.78 (m, 2H), 8.12–8.15 (m, 1H), 8.17–8.20 (m, 1H) ppm; ^13^C NMR (100 MHz, DMSO-*d*_6_): *δ* = 85.35, 96.23, 127.80, 127.95, 128.44, 128.84, 128.92, 129.10, 129.25, 129.54, 130.92, 131.44, 132.35, 132.74, 133.55, 138.22, 142.62, 145.84, 149.12 ppm; MS (EI, 70 eV): *m/z* (%) = 418 ([M^+^][^37^Cl_2_], 12), 416 ([M^+^][^37^Cl][^35^Cl], 53), 414 ([M^+^][^35^Cl_2_], 100), 381 (23), 339 (65), 313 (30), 269 (32), 168 (15); HRMS (ESI): *m/z* = 414.0475.

#### *1*-*(4*-*Methylphenyl)*-*4*-*(2*-*phenylethynyl)[1,2,4]triazolo[4,3*-*a]quinoxaline* (**4j**, C_24_H_16_N_4_)

White solid; yield 288 mg (80 %); m.p.: 218–220 °C; *R*_*f*_ = 0.52; IR (KBr): $\overline{\nu }$ = 2,220, 1,600 cm^−1^; ^1^H NMR (400 MHz, DMSO-*d*_6_): *δ* = 2.39 (s, 3H), 7.36 (d, *J* = 8.0 Hz, 2H), 7.50–7.62 (m, 4H), 7.70–7.77 (m, 3H), 8.08–8.14 (m, 2H), 8.40–8.46 (m, 2H) ppm; ^13^C NMR (100 MHz, DMSO-*d*_6_): *δ* = 22.62, 85.12, 96.21, 116.25, 120.53, 124.62, 126.10, 128.42, 128.60, 129.52, 129.92, 130.62, 132.34, 132.45, 138.40, 140.92, 141.52, 145.22, 149.62 ppm; MS (EI, 70 eV): *m/z* (%) = 360 ([M^+^], 100), 345 (21), 283 (64), 259 (24), 168 (22); HRMS (ESI): *m/z* = 360.1343.

#### *1*-*(4*-*Methoxyphenyl)*-*4*-*(2*-*phenylethynyl)[1,2,4]triazolo[4,3*-*a]quinoxaline* (**4k**, C_24_H_16_N_4_O)

White solid; yield 304 mg (81 %); m.p.: 250–252 °C; *R*_*f*_ = 0.51; IR (KBr): $\overline{\nu }$ = 2,200, 1,620 cm^−1^; ^1^H NMR (400 MHz, DMSO-*d*_6_): *δ* = 3.90 (s, 3H), 7.20 (d, *J* = 8.7 Hz, 2H), 7.41–7.52 (m, 3H), 7.54–7.62 (m, 3H), 7.67–7.70 (m, 1H), 7.75–7.81 (m, 2H), 8.06 (d, *J* = 8.4 Hz, 2H) ppm; ^13^C NMR (100 MHz, DMSO-*d*_6_): *δ* = 55.92, 85.15, 96.20, 116.12, 122.36, 124.51, 126.15, 128.21, 128.42, 128.52, 128.95, 129.55, 129.78, 130.54, 132.32, 132.45, 140.48, 145.23, 149.65 ppm; MS (EI, 70 eV): *m/z* (%) = 376 ([M^+^], 100), 345 (21), 299 (64), 275 (27), 269 (32), 168 (27); HRMS (ESI): *m/z* = 376.1360.
